# Supplementary material for: Improvements in clinical signs of Parkinson’s disease using photobiomodulation: a prospective proof-of-concept study
Source: BMC Neurol. 2021 Jul 2;21:256. doi: 10.1186/s12883-021-02248-y (PMC8249215; doi:10.1186/s12883-021-02248-y)
Supplement: Supplementary file 1 — Additional file 1: Supplementary Table 1. Parameters of the photobiomodulation devices and treatment used in the study. Supplementary Table 2. Mean (st dev) of measures of fine motor control, mobility, balance and cognition before PBM treatment in a second study of PD treatment with PBM. Supplementary Table 3. Individual data for participants before and after treatment with the photobiomodulation protocols. [file 12883_2021_2248_MOESM1_ESM.pdf]

Improvements in clinical signs of Parkinson's disease using photobiomodulation: A prospective proof-of-concept study

Ann Liebert PhD*[1,2], Brian Bicknell PhD[3], E Liisa Laakso PhD[4,5], Gillian Heller PhD[6,7], Parastoo Jalilitabaei MBBS[8], Sharon Tilley BAppSci(Physiotherapy)[9], John Mitrofanis PhD[8], Hosen Kiat[10,11,12]

[1] Department of Medicine and Health Sciences, University of Sydney, Camperdown, Australia

[2] Governance and Research Department, Sydney Adventist Hospital, Wahroonga, Australia

[3] Faculty of Health Sciences, Australian Catholic University North Sydney, Australia

[4] Mater Research Institute, South Brisbane, Australia

[5] Menzies Health Institute Queensland, Griffith University, Gold Coast, Australia

[6] NHMRC Clinical Trials Centre, University of Sydney, Camperdown, Australia

[7] Department of Mathematics and Statistics, Macquarie University, Macquarie Park, Australia

[8] Department of Anatomy, University of Sydney, Camperdown, Australia

[9] Lymphoedema And Laser Therapy, Stepney, Australia

[10] Faculty of Medicine, Health and Human Sciences, Macquarie University, Australia

[11] Faculty of Medicine, University of NSW, Kensington, Australia

[12] Cardiac Health Institute, Sydney, Australia

* correspondence to ann.liebert@sydney.edu.au

Supplementary Table 1. Parameters of the photobiomodulation devices and treatment used in the study.

PARAMETER	SYMBYX PDCARE LASER		VIELIGHT NEURO GAMMA		
Manufacturer	Spectra Analytic Irradia AB		Vielight Inc.		
Diodes	904nm laser diodes (GaAs)		5 x LED diodes		
Wavelength	904nm		810 nm		
Laser class	1		-		
	Clinic laser	Home use laser	posterior	anterior	nasal
Number of diodes	4	3	3	1	1
Output power	30 mW	30 mW	100 mW	75 mW	25mW
Peak power	25,000 mW	25,000 mW			
Pulse frequency	50 Hz	50 Hz	40 Hz	40 Hz	40 Hz
Beam spot size	0.635 cm^2	0.635 cm^2	~1 cm	~1 cm	~1 cm
Power density per diode	47 mW/cm^2	47 mW/cm^2	100 mW/cm^2	75 mW/cm^2	25 mW/cm^2
Total output power	120 mW	60mW	400 mW		
Irradiation time per point	30 s	60 s	2100 s		
Total irradiation time	330 s	660 s	2100 s		
Total energy per point	3.6 J	3.6 J	60 J	45 J	15 J
Number of sites	11 (9 abdomen, 2 neck)	11 (9 abdomen, 2 neck)	3	1	1
Total energy dose per treatment	39.6 J	39.6 J	180 J	45 J	15 J
Treatment frequency weeks 1 to 4	3 x per week for 4 weeks		3 x per week for 4 weeks		
weeks 5 to 8	2 x per week for 4 weeks		2 x per week for 4 weeks		
weeks 9 to 12	1 x per week for 4 weeks		1 x per week for 4 weeks		
weeks 12 to 37 or 52		3 x per week for 25 or 40 weeks	3 x per week for 25 or 40 weeks		

Supplementary Table 2. Mean (st dev) of measures of fine motor control, gait, balance and cognition before PBM treatment in a second study of PD treatment with PBM

	Before treatment
**Gait tests**	
10MWT walk speed (m/sec)	1.69 (0.51) n=7
10MWT stride length (m)	0.97 (0.24) n=7
TUG (s)	7.2 (2.42) n=7
TUG motor (s)	8.1 (1.85) n=7
TUG cognitive (s)	8.4 (2.33) n=7
**Dynamic Balance test**	
Step test - affected leg (n)	15.3 (4.03) n=7
Step test - unaffected leg (n)	16.6 (5.02) n=7
**Cognition test**	
MoCA	25.7 (3.08) n=6
**Fine Motor Skill tests**	
NHPT - affected hand (sec)	29.9 (7.60) n=6
NHPT - unaffected hand (sec)	26.4 (4.79) n=6
Spiral test - dominant hand (sec)	25.6 (9.78) n=6
**Static Balance**	
TS affected leg behind (sec)	14.9 (17.70) n=7
TS unaffected leg behind (sec)	13.0 (10.94) n=7
SLS affected leg raised (sec	7.4 (10.25) n=7
SLS unaffected leg raised (sec)	8.9 (10.59) n=7

10MWT = 10 metre walk test; TUG = Timed-up-and-go; MoCA: Montreal Cognitive Assessment; NHPT = nine-hole peg test; TS = tandem stance (eyes closed); SLS: single leg stance (eyes closed)

Supplementary Table 3. Individual data for participants before and after treatment with the photobiomodulation protocols.

	GROUP AA PARTICIPANTS						GROUP AB PARTICIPANTS					
	AA1	AA2	AA3	AA4	AA5	AA6	AB1	AB2	AB3	AB4	AB5	AB6
**NHPT (seconds)**												
**affected hand**												
12 weeks prior to treatment							29.0	45.8	19.6	25.1	21.0	24.0
immediately before treatment	22.5	23.0	21.2	26.1	21.5	21.6	22.2 (23%)	56.5 -(23%)	19.5 (1%)	31.3 -(24%)	22.3 -(6%)	22.8 (5%)
after 4 weeks treatment (CT)							25.8 (11%)	48.2 -(5%)	21.5 -(10%)	25.7 -(2%)	22.3 -(6%)	19.2 (20%)
after 12 weeks treatment (CT)	21.1 (6%)	28.6 -(24%)	28.0 -(32%)	37.1 -(42%)	17.9 (17%)	27.1 -(25%)	27.9 (4%)	47.6 -(4%)	20.5 -(4%)	34.8 -(38%)	24.1 -(15%)	23.2 (3%)
after 26/38 week home treatm	21.8 (3%)			27.3 -(5%)	21.6 (0%)	29.6 -(37%)	29.4 -(1%)	69.9 -(53%)	22.0 -(12%)	23.4 (7%)	16.4 (22%)	24.4 -(2%)
**dominant hand**												
12 weeks prior to treatment							20.1	30.2	19.6	24.1	21.9	24.0
immediately before treatment	29.3	18.1	33.7	26.4	16.0	25.7	18.0 (11%)	29.1 (4%)	19.5 (1%)	26.6 -(10%)	17.9 (18%)	22.8 (5%)
after 4 weeks treatment (CT)							16.5 (18%)	24.9 (18%)	21.5 -(10%)	26.8 -(11%)	18.7 (15%)	19.2 (20%)
after 12 weeks treatment (CT)	25.8 (12%)	17.9 (1%)	27.7 (18%)	26.8 -(2%)	17.7 -(10%)	20.0 (22%)	25.5 -(27%)	28.3 (6%)	20.5 -(4%)	28.8 -(20%)	20.1 (8%)	23.2 (3%)
after 26/38 week home treatm	21.8 (26%)			27.9 -(6%)	17.0 -(6%)	27.1 -(5%)	17.1 (15%)	29.6 (2%)	20.1 -(2%)	28.8 -(20%)	21.4 (2%)	22.5 (6%)
**SPIRAL TEST (seconds)**												
**dominant hand**												
12 weeks prior to treatment							32.4	37.5	48.6	33.9	29.3	36.5
immediately before treatment	20.4	23.8	29.3	52.5	22.6	26.2	29.6 (9%)	38.2 -(2%)	28.7 (41%)	42.0 -(24%)	20.7 (29%)	40.3 -(10%)
after 4 weeks treatment (CT)							19.4 (40%)	30.8 (18%)	18.7 (61%)	41.7 -(23%)	23.7 (19%)	31.2 (15%)
after 12 weeks treatment (CT)	16.8 (18%)	23.7 (0%)	31.9 -(9%)	23.2 (56%)	16.5 (27%)	21.1 (19%)	28.9 (11%)	29.9 (20%)	29.1 (40%)	45.9 -(35%)	14.1 (52%)	21.7 (41%)
after 26/38 week home treatm	17.8 (13%)	27.6 -(16%)		44.2 (16%)	17.4 (23%)	14.9 (43%)	34.4 -(6%)	34.2 (9%)	19.8 (59%)	35.9 -(6%)	21.1 (28%)	27.9 (24%)
**STEP TEST (number of steps)**												
**affected foot**												
12 weeks prior to treatment							17	11	14	9	9	7
immediately before treatment	12	12	8	14	18	14	21 (24%)	12 (9%)	15 (7%)	13 (44%)	13 (44%)	15 (114%)
after 4 weeks treatment (CT)							23 (35%)	14 (27%)	17 (21%)	15 (67%)	15 (67%)	14 (100%)
after 12 weeks treatment (CT)	15 (25%)	18 (50%)	11 (38%)	20 (43%)	23 (28%)	17 (21%)	23 (35%)	14 (27%)	19 (36%)	11 (22%)	16 (78%)	15 (114%)
after 26/38 week home treatm	15 (25%)	nd	nd	18 (29%)	21 (17%)	17 (21%)	20 (18%)	12 (9%)	21 (50%)	11 (22%)	18 (100%)	14 (100%)
**dominant foot**												
12 weeks prior to treatment							12	10	14	11	12	7
immediately before treatment	12	11	9	12	20	14	18 (50%)	12 (20%)	15 (7%)	11 (0%)	12 (0%)	15 (114%)
after 4 weeks treatment (CT)							21 (75%)	12 (20%)	17 (21%)	15 (36%)	15 (25%)	14 (100%)
after 12 weeks treatment (CT)	14 (17%)	18 (64%)	11 (22%)	16 (33%)	22 (10%)	17 (21%)	21 (75%)	12 (20%)	19 (36%)	13 (18%)	17 (42%)	15 (114%)
after 26/38 week home treatm	14 (17%)	nd	nd	20 (67%)	21 (5%)	17 (21%)	24 (100%)	13 (30%)	21 (50%)	13 (18%)	19 (58%)	14 (100%)
**TS (seconds)**												
**affected foot behind**												
12 weeks prior to treatment							8.0	0.0	2.0	4.0	2.0	0.0
immediately before treatment	30.0	12.0	0.0	0.0	1.0	6.0	8.0 (0%)	1.0	7.5 (275%)	14.0 (250%)	3.6 (80%)	0.0
after 4 weeks treatment (CT)							30.0 (275%)	4.7	30.0 (1400%)	30.0 (650%)	9.0 (350%)	2.7
after 12 weeks treatment (CT)	30.0	30.0 (150%)	9.5	0.0	2.0 (100%)	2.0 -(67%)	15.0 (88%)	1.0	5.6 (180%)	30.0 (650%)	6.0 (200%)	1.0
after 26/38 week home treatm	30.0	nd	nd	0.0	4.0 (300%)	3.9 -(34%)	30.0 (275%)	nd	30.0 (1400%)	3.9 -(2%)	3.4 (70%)	3.0
**unaffected foot behind**												
12 weeks prior to treatment							1.0	0.0	1.0	7.0	4.0	0.0
immediately before treatment	30.0	2.0	0.0	0.0	4.0	7.0	9.2 (820%)	0.0	4.3 (330%)	4.0 -(43%)	13.0 (225%)	0.0
after 4 weeks treatment (CT)							30.0 (2900%)	3.1	3.0 (200%)	30.0 (329%)	3.0 -(25%)	3.3
after 12 weeks treatment (CT)	24.3 -(19%)	30.0 #####	2.3	1.0	3.0 -(25%)	6.0 -(14%)	30.0 (2900%)	2.5	2.0 (100%)	20.0 (186%)	2.0 -(50%)	1.0
after 26/38 week home treatm	30.0 (0%)	nd	nd	0.0	2.7 -(33%)	3.9 -(44%)	30.0 (2900%)	nd	10.7 (972%)	30.0 (329%)	3.4 -(15%)	3.2
**SLS (seconds)**												
**affected foot raised**												
12 weeks prior to treatment							8.9	0.0	0.0	1.0	2.0	2.0
immediately before treatment	6.0	2.0	5.0	0.0	8.0	1.0	2.0 -(78%)	0.0	1.1	3.1 (210%)	2.0 (0%)	1.0 -(50%)
after 4 weeks treatment (CT)							30.0 (237%)	0.1	1.3	1.6 (60%)	0.0	1.0 -(50%)
after 12 weeks treatment (CT)	2.6 -(57%)	14.0 (600%)	0.0	0.0	0.0	2.0 (100%)	10.0 (12%)	0.0	1.6	5.4 (440%)	0.0	0.0
after 26/38 week home treatm	3.9 -(35%)	nd	nd		5.4 -(32%)	1.9 (90%)	23.2 (161%)	nd	5.0	2.1 (110%)	nd	nd
**unaffected foot raised**												
12 weeks prior to treatment							4.9	0.0	0.0	1.0	1.0	1.0
immediately before treatment	5.0	2.0	3.0	1.0	3.0	2.2	12.0 (145%)	0.0	2.1	3.1 (210%)	2.0 (100%)	0.7 -(30%)
after 4 weeks treatment (CT)							6.7 (37%)	0.1	0.1	1.6 (60%)	0.0	0.0
after 12 weeks treatment (CT)	2.4 -(52%)	2.7 (35%)	0.0	12.3 (1130%)	0.0	1.0 -(55%)	26.0 (431%)	0.0	1.7	5.4 (440%)	0.0	0.0
after 26/38 week home treatm	18.9 (278%)	nd	nd		30.0 (900%)	30.0 (1264%)	30.0 (512%)	nd	5.0	2.1 (110%)	nd	nd
**WALK SPEED (metres/second)**												
12 weeks prior to treatment							1.22	1.03	1.62	1.20	1.33	0.76
immediately before treatment	1.00	0.86	0.57	1.03	1.33	1.20	1.76 (44%)	1.54 (50%)	1.88 (16%)	1.94 (61%)	1.90 (43%)	1.50 (98%)
after 4 weeks treatment (CT)							1.94 (58%)	1.62 (58%)	2.00 (23%)	1.94 (61%)	2.04 (53%)	1.55 (104%)
after 12 weeks treatment (CT)	1.54 (54%)	1.62 (89%)	0.79 (39%)	1.67 (61%)	1.94 (45%)	1.71 (43%)	1.94 (58%)	1.60 (56%)	2.21 (36%)	1.94 (61%)	1.96 (47%)	1.50 (98%)
after 26/38 week home treatm	1.72 (72%)	nd	nd	1.33 (29%)	1.75 (32%)	1.09 -(9%)	2.07 (69%)	1.69 (64%)	2.54 (57%)	1.93 (61%)	2.00 (50%)	1.54 (103%)
**WALK STRIDE LENGTH (metres)**												
12 weeks prior to treatment							0.5	0.5	0.5	0.6	0.6	0.5
immediately before treatment	0.5	0.5	0.4	0.5	0.6	0.5	0.6 (20%)	0.7 (33%)	0.8 (38%)	0.9 (43%)	0.6 (0%)	0.7 (33%)
after 4 weeks treatment (CT)							0.4 -(20%)	0.8 (50%)	0.7 (22%)	0.9 (43%)	0.8 (25%)	0.7 (33%)
after 12 weeks treatment (CT)	0.7 (22%)	0.5 (18%)	0.5 (8%)	0.7 (33%)	0.5 -(23%)	0.8 (38%)	0.7 (33%)	0.7 (33%)	0.8 (38%)	0.9 (43%)	0.8 (25%)	0.7 (33%)
after 26/38 week home treatm	0.8 (38%)	nd	nd	0.6 (20%)	0.8 (25%)	0.7 (22%)	0.8 (50%)	0.7 (33%)	0.8 (38%)	0.8 (25%)	0.8 (25%)	0.8 (50%)
**TUG (seconds)**												
12 weeks prior to treatment							7.9	9.7	6.8	7.6	7.8	13.8
immediately before treatment	10.2	8.0	18.0	11.0	6.5	7.5	7.4 (6%)	8.5 (12%)	6.5 (4%)	7.3 (4%)	6.7 (14%)	9.3 (33%)
after 4 weeks treatment (CT)							6.4 (19%)	7.4 (24%)	6.3 (7%)	6.6 (13%)	6.5 (17%)	7.9 (43%)
after 12 weeks treatment (CT)	8.4 (18%)	7.1 (11%)	15.5 (14%)	8.5 (23%)	5.2 (20%)	6.6 (12%)	6.6 (16%)	7.6 (22%)	5.9 (13%)	7.1 (6%)	6.3 (19%)	7.6 (45%)
after 26/38 week home treatm	8.1 (21%)	nd	nd	9.9 (10%)	6.0 (7%)	5.8 (23%)	6.6 (16%)	8.0 (18%)	6.3 (7%)	6.6 (13%)	5.9 (24%)	7.9 (43%)
**TUG MOTOR (seconds)**												
12 weeks prior to treatment							8.8	9.0	6.9	7.6	8.3	11.8
immediately before treatment	10.9	8.2	17.7	10.6	7.1	7.2	8.2 (7%)	8.1 (10%)	6.5 (6%)	8.3 -(9%)	6.4 (23%)	9.1 (23%)
after 4 weeks treatment (CT)							7.4 (16%)	7.2 (20%)	6.6 (4%)	6.9 (9%)	6.4 (23%)	8.3 (30%)
after 12 weeks treatment (CT)	8.8 (19%)	8.2 (0%)	16.5 (7%)	9.3 (12%)	6.6 (7%)	7.0 (3%)	6.4 (27%)	8.5 (6%)	6.3 (9%)	7.1 (7%)	6.6 (20%)	8.1 (31%)
after 26/38 week home treatm	8.4 (23%)	nd	nd	13.3 -(26%)	6.1 (14%)	6.4 (11%)	6.0 (32%)	9.2 -(2%)	7.0 -(1%)	6.8 (10%)	5.8 (30%)	8.4 (29%)
**TUG COGNITIVE (seconds)**												
12 weeks prior to treatment							10.3	10.6	7.2	8.7	8.8	13.8
immediately before treatment	12.0	10.4	23.7	10.5	6.0	7.6	7.5 (27%)	8.8 (17%)	7.3 -(1%)	7.5 (14%)	6.7 (24%)	9.0 (35%)
after 4 weeks treatment (CT)							7.2 (30%)	8.2 (23%)	6.7 (7%)	6.6 (24%)	6.2 (30%)	8.7 (37%)
after 12 weeks treatment (CT)	9.3 (23%)	6.9 (34%)	18.2 (23%)	9.3 (11%)	5.2 (14%)	6.8 (11%)	6.3 (39%)	8.9 (16%)	6.4 (11%)	6.8 (22%)	6.8 (23%)	8.1 (41%)
after 26/38 week home treatm	8.8 (26%)	nd	nd	9.8 (6%)	5.2 (13%)	5.9 (22%)	6.7 (35%)	9.5 (11%)	6.5 (10%)	6.4 (27%)	6.6 (25%)	8.8 (36%)
MoCA score												
12 weeks prior to treatment							26	28	27	26	24	21
immediately before treatment	24	26	26	28	27	24	29 (12%)	29 (4%)	27 (0%)	26 (0%)	28 (17%)	24 (14%)
after 4 weeks treatment (CT)							28 (8%)	30 (7%)	28 (4%)	26 (0%)	28 (17%)	25 (19%)
after 12 weeks treatment (CT)	25 (4%)	26 (0%)	28 (8%)	28 (0%)	27 (0%)	29 (21%)	29 (12%)	30 (7%)	27 (0%)	27 (4%)	29 (21%)	29 (38%)
after 26/38 week home treatm	29 (21%)	29 (12%)	nd	30 (7%)	28.7 (6%)	30 (24%)	30 (15%)	30 (7%)	30 (11%)	28.7 (10%)	30 (25%)	nd

NHPT = nine-hole peg test
TS = tandem stance (eyes closed)
SLS = single leg stance (eyes closed)
TUG = timed up-and-go
MoCA = Montreal Cognitive Assessment
number in brackets is the percentage improvement (positive value) or decline (negative value) in an outcome measure
